# A Novel Transgenic Mouse Model Implicates *Sirt2* as a Promoter of Hepatocellular Carcinoma

**DOI:** 10.3390/ijms241612618

**Published:** 2023-08-09

**Authors:** Alexandra V. Schmidt, Satdarshan P. Monga, Edward V. Prochownik, Eric S. Goetzman

**Affiliations:** 1 Division of Genetic and Genomic Medicine, Department of Pediatrics, University of Pittsburgh School of Medicine, University of Pittsburgh, Pittsburgh, PA 15260, USA; 2 Pittsburgh Liver Research Center, University of Pittsburgh Medical Center, Pittsburgh, PA 15213, USA; 3 Division of Experimental Pathology, Department of Pathology, University of Pittsburgh School of Medicine, Pittsburgh, PA 15213, USA; 4 Division of Gastroenterology, Hepatology and Nutrition, Department of Medicine, University of Pittsburgh School of Medicine, Pittsburgh, PA 15213, USA; 5 Division of Hematology and Oncology, Department of Pediatrics, University of Pittsburgh School of Medicine, University of Pittsburgh, Pittsburgh, PA 15260, USA

**Keywords:** sirtuin-2 (*Sirt2*), hepatocellular carcinoma (HCC), c-MYC, liver, transgenic

## Abstract

Hepatocellular carcinoma (HCC) is one of the leading causes of cancer deaths globally. Incidence rates are steadily increasing, creating an unmet need for new therapeutic options. Recently, the inhibition of sirtuin-2 (*Sirt2*) was proposed as a potential treatment for HCC, despite contradictory findings of its role as both a tumor promoter and suppressor in vitro. *Sirt2* functions as a lysine deacetylase enzyme. However, little is known about its biological influence, despite its implication in several age-related diseases. This study evaluated *Sirt2*’s role in HCC in vivo using an inducible *c-MYC* transgene in *Sirt2^+^*^/*+*^ and *Sirt2*^−/−^ mice. *Sirt2*^−/−^ HCC mice had smaller, less proliferative, and more differentiated liver tumors, suggesting that *Sirt2* functions as a tumor promoter in this context. Furthermore, *Sirt2*^−/−^ HCCs had significantly less c-MYC oncoprotein and reduction in c-MYC nuclear localization. The RNA-seq showed that only three genes were significantly dysregulated due to loss of *Sirt2*, suggesting the underlying mechanism is due to *Sirt2*-mediated changes in the acetylome, and that the therapeutic inhibition of *Sirt2* would not perturb the oncogenic transcriptome. The findings of this study suggest that *Sirt2* inhibition could be a promising molecular target for slowing HCC growth.

## 1. Introduction

Liver cancer was the fourth leading cause of cancer deaths globally in 2020 [1]. Hepatocellular carcinoma (HCC) is the most common form of primary liver cancer, and its incidence has been increasing since the 1980s, carrying a significant global health burden [2,3]. HCC has a complex, heterogeneous disease etiology, typically requiring underlying liver cirrhosis associated with conditions such as chronic hepatitis B virus (HBV) or hepatitis C virus (HCV) infection, alcoholism, diabetes, and non-alcoholic fatty liver disease (NAFLD) [4]. Epidemiological trends indicate that the rising incidence of HCC is related to the underlying causes of liver cirrhosis, especially obesity and NAFLD, which are also becoming increasingly prevalent [4,5]. Clinical management of HCC depends on tumor size, patient comorbidities, and the severity of the underlying liver disease. The best treatment option for HCC is orthotopic liver transplantation (OLT), and the 5-year survival rates post-surgical intervention are typically >70% [6]. Unfortunately, a tumor burden beyond which transplant becomes contraindicated exists, and many patients become ineligible for this procedure while awaiting it. This creates an unmet need for new treatment options that slow tumor growth and extend the transplant window [4,7], spurring research into a host of alternative therapies such as Traditional Chinese medicine, herbal medicines, gene therapy, and photothermal therapy [8,9,10].

A vast majority of HCC cases include upregulation of the oncogene *c-MYC*, which manifests as a more advanced, aggressive, and fast-growing tumor phenotype [11,12]. Therapeutic strategies to inhibit c-MYC function and its downstream targets have been attempted but ultimately failed due to c-MYC’s ‘undruggable’ structure and short half-life [13,14,15]. Therefore, targeting factors that regulate c-MYC expression or stability may be attractive options. One such factor may be sirtuin-2 (*Sirt2*) [16,17,18]. Recently, small molecule inhibition of *Sirt2* has been identified as a potential therapeutic strategy for a wide variety of cancers, including HCC [17,19,20]. *Sirt2*, which specifically de-acetylates lysine residues, is unique because it is the only member of the sirtuin family that is localized to both the cytosolic and nuclear compartments [21]. When the *Sirt2* literature is taken as a whole, two common threads emerge: (1) *Sirt2* serves a role in the nucleus regulating the cell cycle; and (2) *Sirt2* regulates glucose metabolism in the cytosol. Loss of cell cycle regulation and altered cell metabolism are two imperative hallmarks of cancer; therefore, *Sirt2* may represent an “Achilles’ heel” that could simultaneously affect both of these key tumorigenic phenotypes. Treatment of a broad range of cancer cell lines with Thiomyristoyl (TM) or RK-9123016, which are potent, selective *Sirt2* inhibitors that slow cell migration, sphere formation, and reduce cancer cell viability [16,17]. Notably, inhibiting *Sirt2* reduces the abundance of *c-MYC* due to effects on MYC stability [16].

While several in vitro studies point to *Sirt2* inhibition as a promising therapeutic target for HCC, some in vivo data counters this notion. Firstly, male *Sirt2*^−/−^ mice develop liver tumors with advanced age, suggesting that *Sirt2* may suppress rather than promote HCC [22,23]. Secondly, human HCCs have lower levels of *SIRT2* expression compared to adjacent tissue [23]. However, additional studies that queried The Cancer Genome Atlas (TCGA) further showed that *SIRT2* expression in HCCs directly correlate with worse patient survival, prognosis, and increased microscopic vascular invasion [24,25]. In human breast cancer, mixed roles for *Sirt2* have also been observed, with grade 1 tumors displaying decreased *Sirt2* expression and higher-grade tumors showing elevated expression [26]. It may very well be possible that *Sirt2* “flips” from being a tumor suppressor (via nuclear effects on the cell cycle) to a tumor promoter after transformation (via cytosolic effects on metabolism). In the present study, we asked whether the presence of *Sirt2* slows or enhances tumor growth in a *c-MYC*-driven mouse model of HCC.

## 2. Results

### 2.1. Optimization of a Transgenic HCC Mouse Model

**Figure 1:**
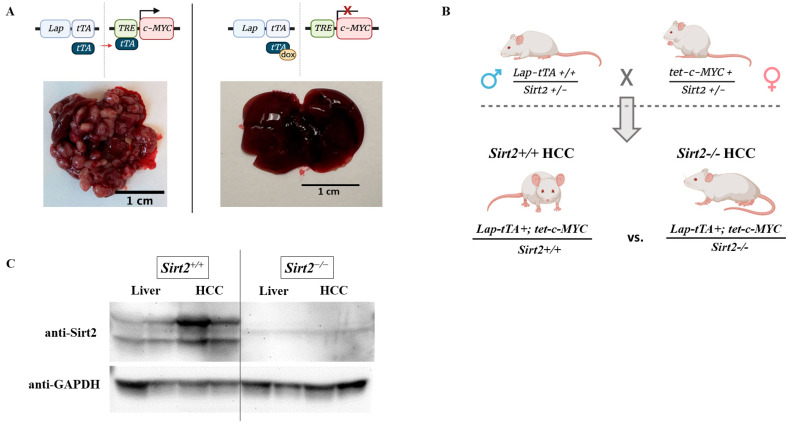
(**A**) HCC-like tumors develop in mice harboring a c-MYC transgene. The liver promotor, Lap, drives tTA expression in hepatocytes. The product of tTA is a transcription factor that binds to its response element, TRE, to drive downstream c-MYC expression. (**B**) Males homozygous for Lap-tTA and heterozygous for *Sirt2* are crossed with females hemizygous for the Tet-c-MYC transgenes and heterozygous at the *Sirt2* allele to produce offspring with two distinct tumor phenotypes: *Sirt2*^+/+^ HCC and *Sirt2*^−/−^ HCC. Due to this breeding strategy, littermate controls(*Sirt2^+^*^/*+*^ liver and *Sirt2*^−/−^) with a normal liver phenotype were also generated for normal liver comparison, used throughout the manuscript. (**C**) *Sirt2* is present in both normal liver and HCC liver samples from this colony. *Sirt2* is not present in *Sirt2*^−/−^ control livers nor *Sirt2*^−/−^ HCC tumors.

HCC was modeled using a tetracycline-suppressible liver-specific *c-MYC* transgene (Figure 1A) [27]. This transgenic model was adapted to study the loss of *Sirt2* in HCC by crossing it with a global *Sirt2* knockout mouse (*Sirt2*^−/−^). To minimize potential artifacts caused by a mix of genetic backgrounds, *Sirt2^+^*^/−^ mice hemizygous for the tetracycline-repressible *c-MYC* transgene were crossed with *Sirt2^+^*^/−^ mice homozygous for the tetracycline transactivator (*tTA*) transgene (Figure 1B). Subsequent litters were screened to identify two groups, each of control and experimental mice: (1) *Sirt2^+^*^/*+*^ or *Sirt2*^−/−^ mice hemizygous for both transgenes (hereafter, *Sirt2^+^*^/*+*^ HCC or *Sirt2*^−/−^ HCC); and (2) *Sirt2^+^*^/*+*^ or *Sirt2*^−/−^ mice negative for the *tet-MYC* transgene (*Sirt2^+^*^/*+*^ or *Sirt2*^−/−^ with a normal liver phenotype), serving as tumor-free controls. Breeding pairs and neonatal mice were maintained on 0.1 mg/mL doxycycline drinking water to suppress *c-MYC* expression throughout development. All of the studies described here used the removal of doxycycline at 28 days of age to induce tumorigenesis, a time point optimized through pilot studies. Western blot data show that two isoforms of *Sirt2* were present in normal liver and tumor homogenates prepared from *Sirt2^+^*^/*+*^ mice; however, *Sirt2* protein was not present in either the normal or disease liver homogenates prepared from *Sirt2*^−/−^ mice (Figure 1C).

### 2.2. Sirt2 Deficiency Produces Morphologically Distinct HCCs

**Figure 2:**
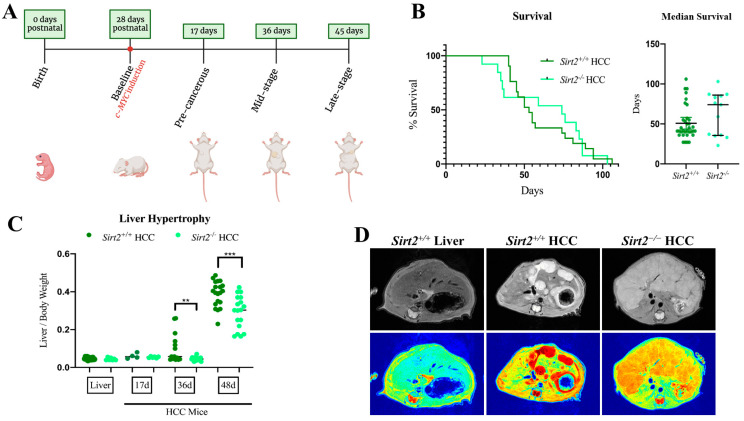
(**A**) c-MYC expression is induced via doxycycline removal from drinking water 28 days postnatal. c-MYC expression results in liver tumors that represent pre-, mid-, and late-stage HCC progression at different time points. (**B**) Survival analysis of *Sirt2*^−/−^ (*n* = 14) vs. *Sirt2*^+/+^ (*n* = 22) HCC shows that there is no significant difference in survival (*p*-value = 0.9705). Median survival days (error bars represent IQR) were not significantly different (*p*-value = 0.1100). (**C**) Liver/body weight ratios 17, 36, and 48-days post c-MYC induction in *Sirt2*^−/−^ HCC vs. *Sirt2*^+/+^ HCC mice. *Sirt2*^−/−^ mice have significantly reduced liver/body weight ratios 36 (*n* = 14) and 48 (*n* = 20) days post-c-MYC induction. ** *p* < 0.01; *** *p* < 0.001. (**D**) Representative images of MRIs taken 36 days post-c-MYC induction. Shown in the first panel is a normal liver from a non-HCC wildtype mouse as a visual comparison. Addition of a filter that aids in tissue density visualization (red = more dense, blue = less dense) suggests that *Sirt2*^−/−^ HCC mice have smaller, more dispersed tumor nodes compared to the distinctive, dense tumors in *Sirt2*^+/+^ HCC mice. A total of *n* = 4 *Sirt2*^−/−^ HCC mice and *n* = 3 *Sirt2*^+/+^ HCC mice were imaged with similar results.

An initial cohort of *Sirt2^+^*^/*+*^ HCC and *Sirt2*^−/−^ HCC mice were observed for overall differences in disease progression. Knocking out *Sirt2* did not statistically change the overall survival time, as both groups had succumbed to the disease by ~105 days post-*c-Myc* induction (Figure 2B). However, the median survival time was 75 days for *Sirt2*^−/−^ versus 41 days for *Sirt2^+^*^/*+*^ (Figure 2B), suggesting that *Sirt2* does indeed accelerate HCC progression. Next, we used pilot data of MYC expression levels and liver histology in *Sirt2^+^*^/*+*^ HCC mice to establish 17-, 36-, and 48-days post-*c-MYC* induction as time points that replicate early-, mid-, and late-stage disease, respectively (Figure 2A). At 17 days, tumors in *Sirt2^+^*^/*+*^ livers were just initiating. At 36 days, tumor growth had increased, but had not reached lethality. By 48 days, about 50% of *Sirt2^+^*^/*+*^ HCC mice had succumbed to the HCC phenotype. This was supported by liver:body weight ratios, which increased dramatically between 36 and 48 days (Figure 2C). This rapid expansion phase appeared to be delayed in *Sirt2*^−/−^ HCC mice. At 36 days, the liver:body weight ratios remained unchanged from baseline, and were significantly lower than those observed in *Sirt2^+^*^/*+*^ HCC mice. As seen in the survival curve, this time point corresponded with a period of quiescence, during which mortality leveled off in the *Sirt2*^−/−^ HCC mice. This suggested that *Sirt2*^−/−^ HCC tumors had restricted growth once they progressed past an early-stage disease time point. To view these changes in vivo, a cohort of *Sirt2*^−/−^ HCC and *Sirt2^+^*^/*+*^ HCC mice was subjected to serial MRI scans 36 days post-*c-MYC* induction, and a jet overlay was applied to each image for interpretation of tissue density. Visible tumors were present in both genotypes. However, *Sirt2*^−/−^ HCC livers appeared to contain more diffuse, less dense tumor nodules than *Sirt2^+^*^/*+*^ HCC livers (Figure 2D).

**Figure 3:**
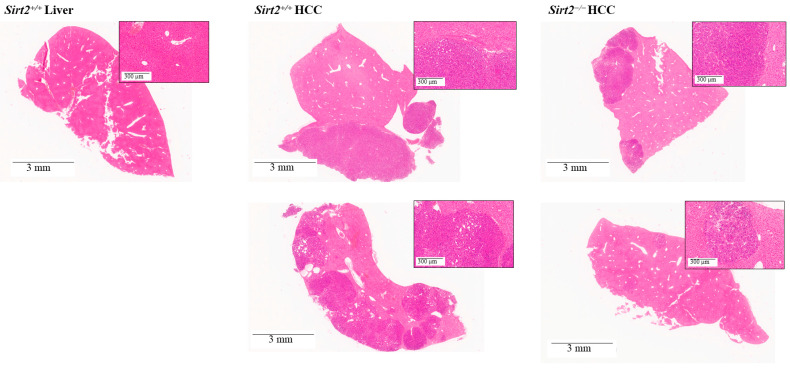
Whole-slide scans of liver tissue stained with H&E from *Sirt2^+^*^/*+*^ HCC and *Sirt2*^−/−^ HCC mice 36 days post-*c-MYC* induction indicate that *Sirt2*^−/−^ HCC mice have different liver tissue architecture than *Sirt2^+^*^/*+*^ HCC mice. Pink areas indicate normal tissue and purple areas indicate tumors. *Sirt2*^−/−^ HCC livers have smaller and fewer purple areas than *Sirt2^+^*^/*+*^ HCC livers. Shown in the first panel is a normal liver from a non-HCC wildtype mouse as a visual comparison.

At this same 36-day time-point, whole-slide scans of liver segments stained with hematoxylin and eosin (H&E) confirmed that *Sirt2*^−/−^ HCC mice had much smaller areas of neoplasia (areas of purple stain) compared to *Sirt2^+^*^/*+*^ HCC mice (Figure 3).

### 2.3. Loss of Sirt2 Results in Lower Tumor Grade

**Figure 4:**
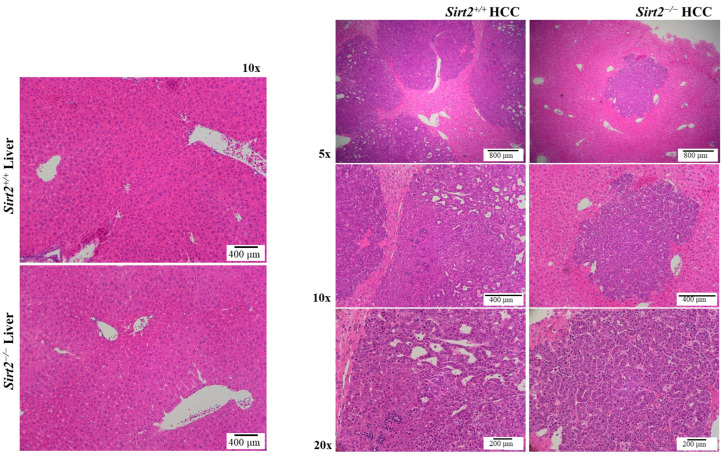
Histopathological interpretation showed that *Sirt2*^−/−^ tumor cells are more differentiated than *Sirt2^+^*^/*+*^ tumor cells. At 20× magnification, *Sirt2^+^*^/*+*^ HCC tumors have more capillary recruitment and less organization around these structures, suggesting loss of portal triad structures found in normal liver. These structures are formed by normal hepatocytes. *Sirt2^+^*^/*+*^ and *Sirt2*^−/−^ livers shown for visual representation.

With histopathological interpretation of the H&E slides, we determined that the tumor tissue architecture of *Sirt2*^−/−^ HCC tumors was indeed different from that of *Sirt2^+^*^/*+*^ HCC tumors (Figure 4). Specifically, the cells comprising *Sirt2*^−/−^ HCCs were more differentiated than those comprising *Sirt2^+^*^/*+*^ HCCs. Greater cell type differentiation is typically associated with better survival rates in solid tumors [28,29]. At 20× magnification, *Sirt2^+^*^/*+*^ HCCs had more capillary recruitment and less organization around these structures, suggesting the loss of portal triad structures found in normal liver. These structures are formed by normal hepatocytes and are required for normal liver function.

**Figure 5:**
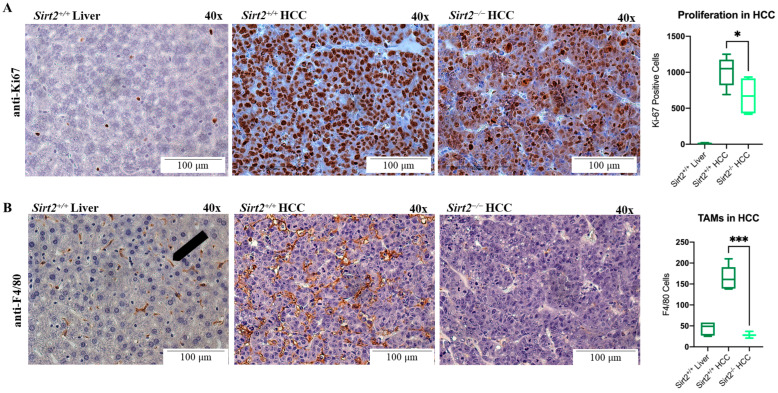
Magnification at 40× of *Sirt2*^+/+^ liver, *Sirt2*^+/+^ HCC, and *Sirt2*^−/−^ HCC tissues immunostained with anti-Ki67 and anti-F4/80. (**A**) Quantification of *Sirt2*^+/+^ HCC and *Sirt2*^−/−^ HCC tissue indicates that *Sirt2*^−/−^ HCC tumors have significantly fewer Ki-67 positive cells (*n* = 25). (**B**) *Sirt2*^−/−^ HCC tumors have significantly fewer TAMs than *Sirt2*^+/+^ HCC tumors (*n* = 25). * *p* < 0.05; *** *p* < 0.001.

Quantification of Ki-67 positive cells showed that *Sirt2*^−/−^ HCC tumors were significantly less proliferative than *Sirt2^+^*^/*+*^ HCCs (Figure 5A). Quantification of tumor-associated macrophages (TAMs) showed that *Sirt2*^−/−^ HCCs had significantly fewer TAMs than *Sirt2^+^*^/*+*^ HCCs (Figure 5B). TAMs are typically a marker for tumor grade—where higher numbers of TAMs are indicative of a tumor-supportive microenvironment and a worse prognosis [30]. Since *Sirt2*^−/−^ HCCs were more differentiated and had fewer proliferative and immunological markers than *Sirt2^+^*^/*+*^ HCCs, a loss of *Sirt2* resulted in a lesser HCC tumor grade 36 days post-*c-MYC* induction.

### 2.4. Loss of Sirt2 Reduces c-MYC Levels and Nuclear Localization

**Figure 6:**
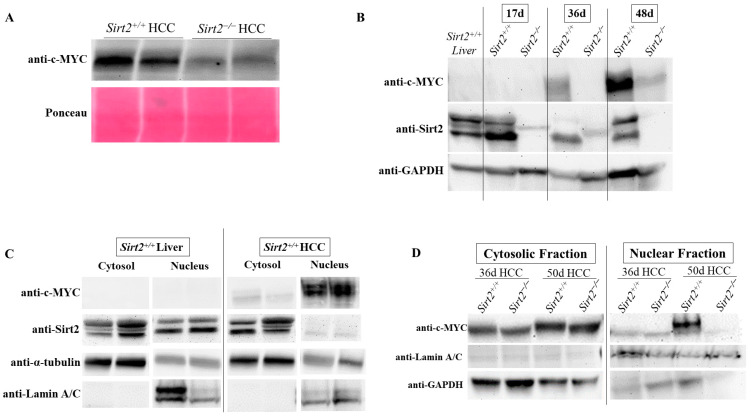
(**A**) *Sirt2*^−/−^ HCC tumor homogenate has less c-MYC than *Sirt2^+^*^/*+*^ HCC tumor homogenate. (**B**) In normal liver, *Sirt2* is localized to both the nucleus and cytosol. In tumors, *Sirt2* is predominantly localized to the cytosol and not to the nucleus, suggesting it is mostly interacting with cytosolic targets in tumors. Due to its function as a transcription factor, c-MYC is mostly localized to the nucleus in tumors. (**C**) c-MYC is present in both the nucleus and cytosol of *Sirt2^+^*^/*+*^ HCC mice. (**D**) Comparison of *Sirt2^+^*^/*+*^ and *Sirt2*^−/−^ HCCs showed c-MYC was not present in the nuclear fraction of *Sirt2*^−/−^ HCC tumors, suggesting that *Sirt2* is necessary for c-MYC nuclear translocation. These bands were re-arranged for clarity and brevity.

To begin investigating a potential mechanism behind the smaller, less developed tumors in *Sirt2*^−/−^ HCC mice, the amount of c-MYC in *Sirt2*^−/−^ vs. *Sirt2^+^*^/*+*^ HCCs was measured. c-MYC has been shown to be acetylated in vitro, and previous studies suggest that *Sirt2* directly affects the stability of c-MYC through acetylation and the recruitment of ubiquitin groups, which targets c-MYC for degradation by ubiquitin E3 ligase [31]. Indeed, *Sirt2*^−/−^ HCC tumors had significantly less steady state levels of c-MYC oncoprotein than *Sirt2^+^*^/*+*^ HCC mice (Figure 6A). Since both c-MYC and *Sirt2* localize to the nucleus, nuclear and cytosolic fractions from *Sirt2^+^*^/*+*^ liver and *Sirt2^+^*^/*+*^ HCC tissue were prepared and probed for their compartmental c-MYC and *Sirt2*. *Sirt2* was present in both the cytosol and nucleus of normal liver tissue but was mostly cytosolic in HCC tumors (Figure 6B). Furthermore, c-MYC was mostly located in the nucleus of HCC tissues, which is consistent with its function as a transcription factor.

In an advanced HCC phenotype 50 days post-*c-MYC* induction, c-MYC was present mostly in the nucleus of *Sirt2*^+/+^ HCCs (Figure 6C). It is important to note that both fractions were exposed on the same membrane, likely causing the high intensity of the nuclear HCC samples to washout the less intense signals from the cytosolic HCC samples. *Sirt2* localization was the opposite, where the cytosolic fraction contained a vast majority of *Sirt2* (Figure 6C). To better evaluate the compartmental differences in c-MYC abundance due to loss of *Sirt2* in vivo, nuclear and cytosolic fractions were prepared from *Sirt2^+^*^/*+*^ and *Sirt2*^−/−^ HCCs (Figure 6D). The fractions were subject to immunoblotting and exposure on separate membranes to avoid the nuclear c-MYC signal from potentially washing out the cytosolic c-MYC signal (as seen in Figure 6C). Cytosolic preparations of both *Sirt2^+^*^/*+*^ and *Sirt2*^−/−^ HCCs show relatively similar amounts of c-MYC, but only an increased amount of c-MYC was found in the *Sirt2^+^*^/*+*^ HCC nuclear compartment 50-days post-c-MYC induction. With the localization of *Sirt2* in the cytosol of *Sirt2^+^*^/*+*^ HCCs, this suggested that a vast majority of what minimal c-MYC was present in the *Sirt2*^−/−^ HCCs was sequestered in the cytosol due to the loss of *Sirt2*.

### 2.5. The HCC Transcriptome Is Not Altered by the Loss of Sirt2

**Figure 7:**
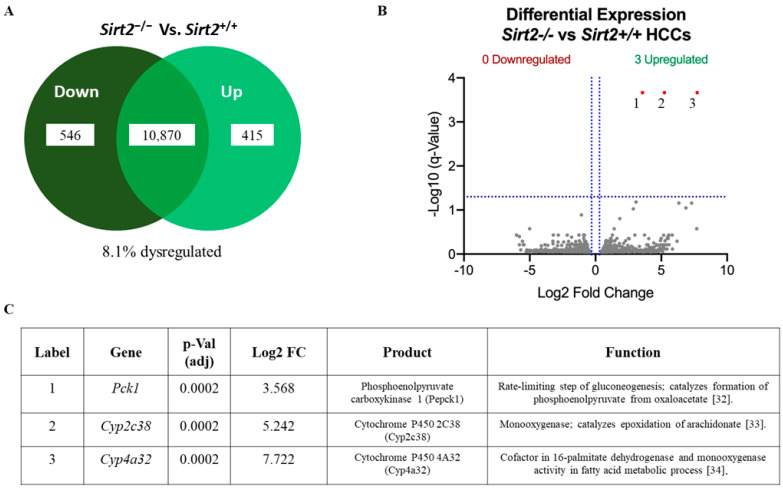
(**A**) Of 11,381 total genes, 8.1% are dysregulated in *Sirt2*^−/−^ HCC mice before controlling for false discovery rate (*p*-value ≤ 0.05; *n* = 4). See Table S1 for a list of these genes. (**B**) When false discovery rate is controlled for, only 3 genes are significantly upregulated (q-value < 0.01), indicated with numbers 1–3 on the graph. These three upregulated genes are listed in panel (**C**) in order of increasing log2 fold change, with gene product annotation [32,33,34].

The above observations led us to theorize that *Sirt2* may influence the nuclear translocation of c-MYC, and thus its function as a transcription factor. To test this, we employed RNA-seq to profile HCC tumor tissue from *Sirt2^+^*^/*+*^ and *Sirt2*^−/−^ mice. Contrary to expectations, eliminating *Sirt2* did not alter the expression of c-MYC target genes. In fact, when controlling for false discovery by restricting the confidence interval to a q-value < 0.01, only three genes were significantly upregulated in *Sirt2*^−/−^ HCCs, and none were downregulated (Figure 7B). One upregulated gene, *Pck1*, encodes the rate-limiting enzyme of gluconeogenesis, phosphoenolpyruvate carboxykinase. Two members of the cytochrome P450 (CYP) family, *Cyp2c38* and *Cyp4a32*, were also increased, and both are involved in the cellular clearance of xenobiotics. Importantly, *c-MYC* was not identified as being significantly dysregulated at the mRNA level, indicating that the effect of *Sirt2* on c-MYC abundance (see Figure 6) was likely a post-transcriptional one. Likewise, the lack of transcript changes overall indicated that any effect of *Sirt2* on tumor growth was probably occurring at a post-transcriptional level, in accordance with its role as a lysine deacetylase.

## 3. Discussion

Evaluation of a novel transgenic mouse model of HCC provides evidence that *Sirt2* functions as a tumor promotor in vivo. *Sirt2*^−/−^ HCC mice had smaller, less proliferative, and more differentiated liver tumors, suggesting that *Sirt2* is permissive for tumorigenesis and promotes the development of HCC. A major finding from this study indicated that *Sirt2* loss significantly decreased the level of c-MYC in vivo. This finding confirms previous reports that *Sirt2* inhibition affects c-MYC stability and function in vitro [16,31]. To further our understanding of this mechanism, we found significantly less nuclear c-MYC in tumor tissue derived from *Sirt2*^−/−^ HCC mice, suggesting that c-MYC is targeted for degradation prior to entering the nuclear compartment where it functions as a transcription factor. Additional investigations will be required to elucidate the mechanisms by which *Sirt2* delays *c-MYC*-driven HCC tumorigenesis in vivo, possibly through a regulatory pathway involving ubiquitination as previously reported [16]. Investigative questions include whether *Sirt2* and c-MYC directly interact in the cytosol and/or nucleus, if hyperacetylation alone decreases c-MYC activity, whether the loss of *Sirt2* is requisite in this mechanism, and if inhibiting it has the potential to be exploited as a therapeutic option.

Significantly fewer TAMs were present in *Sirt2*^−/−^ HCCs, representing a novel finding that suggests that a global loss of *Sirt2* is detrimental to either immune escape or compromised immune surveillance. TAMs are typically sequestered in high numbers to create an immunosuppressive tumor microenvironment by releasing cytokines that inhibit immune checkpoint proteins released by T-cells [30,35]. It was unclear from our study design whether fewer TAMs were caused by a lower tumor grade in *Sirt2*^−/−^ HCCs, or if the immune system in *Sirt2*^−/−^ HCC mice (and *Sirt2*^−/−^ mice in general) was somehow compromised. There is some evidence to suggest that *Sirt2* regulates the tumor microenvironment by suppressing regulatory T-cells, promoting invasion through inhibition of the E-cadherin pathway, and increasing the acidity of surrounding tissues [24,36,37,38]. This could have translational relevance, as one recent study showed *SIRT2* expression to be significantly lower in peripheral T-lymphocytes of breast cancer patients, which suggested insufficient antitumor immunity due to compromised detection of tumor cells [39]. Tumor cells rely on escaping immune surveillance to continuously grow and develop; therefore, it is critical to better understand how *Sirt2* not only affects the tumor microenvironment, but whether it plays a role in the normal immune response, and if this role is intrinsic to the immune system or to the tumor of origin.

Our study is the first to use a transgenic mouse model while controlling for the possibility that *Sirt2* functions as both a tumor promotor and suppressor in vivo, which proved to be scientifically relevant, as the MRI data and histopathological investigation in our study indicate stark phenotypic differences due to the sustained loss of *Sirt2*. We surmised that this hypothesis of a “flip” in the role of *Sirt2*, from tumor suppressor in a normal liver context to tumor promotor in a tumor context, could be applied to the HCC literature, as female *Sirt2*^−/−^ knockout mice in a controlled environment develop mammary or liver tumors, but inhibition of *Sirt2* in well-established cancer cell lines suggest a tremendous therapeutic effect. Altogether, these data suggest that *Sirt2* ablation is detrimental to tumor growth, which supports previous in vitro studies that showed *Sirt2* functions as a tumor promotor [24,25,40]. Ultimately, we concluded that *Sirt2* is a tumor promotor, but only in an in vivo HCC context. More research will need to be carried out to determine whether *Sirt2* is a tumor suppressor, as data suggest, in a normal liver context. Furthermore, more investigative research into *Sirt2*’s function in a pre-HCC state, such as in a fibrotic or cirrhotic liver, must be conducted.

A possible mechanism to explain the profound difference in HCC phenotypes due to a loss of *Sirt2* could be in aerobic glycolysis, or the Warburg effect, which is necessary to support tumor growth and cellular proliferation; a loss of *Sirt2* could inhibit maximal cellular respiration. This has previously been suggested, since *Sirt2* is necessary for the stability and function of tumor-specific pyruvate kinase isoform 2 (Pkm2) [24]. We further suspect that a loss of nuclear MYC caused by the loss of *Sirt2* reduces the expressions of genes essential to the Warburg effect, such as Pkm2, glyceraldehyde-3-phosphate dehydrogenase (Gapdh), and lactate dehydrogenase A (Ldha) [41,42]. It is possible that *Sirt2* supports normal biological processes, likely through stabilizing chromatin and cell cycle regulatory complexes, in normal tissue contexts [20,23,25]. However, once control of the cell cycle is lost in cancerous cells, *Sirt2* stays in the cytosol (as Figure 6B suggests) and supports the cell’s reliance on aerobic glycolysis.

Some major limitations pertaining to this study involve the use of the *c-MYC* inducible HCC model. The expression of *c-MYC* was suppressed through the administration of doxycycline in the water. HCC breeders were maintained with water containing doxycycline throughout the embryonic and neonatal stages, so pups only had access to doxycycline through the mother. Ideally, each pup would have been given the exact same amount of doxycycline, but that proved to be a difficult variable to control due to doxycycline stability, as others have previously reported with doxycycline administration via drinking water [43]. Similarly, variations in water intake by the mothers could alter the efficacy of *c-Myc* suppression during embryonic development. Furthermore, the use of a liver conditional *Sirt2* knockout mouse would provide better insights into the observed whole-body phenotype; specifically, the observation of fewer TAMs in *Sirt2*^−/−^ HCC. Whether this potential escape from immunosurveillance is due to lack of *Sirt2* in the tissue of origin or lack of *Sirt2* in the immune system is unclear. An immunopanel and/or use of macrophage knockout mice would provide more insights into this potentially exciting mechanism of *Sirt2*-mediated immunosuppression, suggested by a recent study that showed *Sirt2* affects the metabolic fitness of T-cells in the microenvironment of patients with advanced non-small cell lung cancer [44]. Our previous research has shown that a mouse liver tumor model of another form of liver cancer, hepatoblastoma (HB), required c-MYC for HBs to achieve maximal tumor growth [45]. Along with the evidence from this research, c-MYC could be requisite in maintaining tumor growth and burden, further supporting the notion of its inhibition as a therapeutic strategy in primary liver cancer.

## 4. Materials and Methods

### 4.1. Animals

The University of Pittsburgh IACUC approved all animal protocols, and all experiments were conducted in accordance with the guidelines and regulations set forth in the AWA and PHS Policy on Humane Care and Use of Laboratory Animals. All of the mice were maintained on a 12 h light/dark cycle in a pathogen-free barrier facility. The *Sirt2*Tm1.1Fwa (*Sirt2*^−/−^) mice on the C57BL/6J strain were sourced from Jackson Laboratories (Bar Harbor, ME). The HCC was modeled in a murine liver using the well-studied conditional mouse model that harbors a tetracycline-suppressible *c-Myc* transgene. The colony relies on breeders—FVB/N-Tg(*tetOMYC*)36aBop/J females (termed the “*TRE-MYC* line”) and B6.Cg-Tg(*Cebpb-tTA*)5Bjd/J males (termed the “*LAP-tTA*” line)—to generate both male and female offspring that develop liver tumors upon removal of doxycycline from the water [46]. The breeders to initiate the colony were kindly provided by the Prochownik lab [41]. To ascertain the data regarding *Sirt2* ablation in HCC, *Sirt2^+^*^/−^ mice were bred with both the *TRE-MYC* and *LAP-tTA* lines to generate new HCC colony breeders that were heterozygous at the *Sirt2* locus. Subsequent litters were screened to identify two groups, each of control and experimental mice: (1) *Sirt2^+^*^/*+*^ or *Sirt2*^−/−^ mice hemizygous for both transgenes (hereafter, *Sirt2^+^*^/*+*^ HCC or *Sirt2*^−/−^ HCC); and (2) *Sirt2^+^*^/*+*^ or *Sirt2*^−/−^ mice negative for the *tet-MYC* transgene (*Sirt2^+^*^/*+*^ or *Sirt2*^−/−^ with a normal liver phenotype), serving as tumor-free controls. This generated male and female experimental mice and minimized potential artifacts caused by a mix of background genetics. The breeding pairs and neonatal mice were maintained on 0.1 mg/mL doxycycline drinking water to suppress *c-MYC* expression throughout development [36]. Doxycycline was removed from the water of all animals at 28 days of age to induce tumorigenesis—a time point optimized through pilot studies. All of the mice and breeders were maintained in-house.

### 4.2. Tumor Characterization

A survival curve was generated by allowing a cohort of *Sirt2^+^*^/*+*^ HCC and *Sirt2*^−/−^ HCC of both sexes to progress to end-stage disease, defined as the point when HCC mice ceased grooming and showed significantly decreased mobility due to abdominal distension. This behavior indicated that death was imminent within 24 h; the survival time was defined as the number of days between doxycycline removal and sacrifice. The *Sirt2^+^*^/*+*^ and *Sirt2*^−/−^ HCC mice that were 17-, 36-, and 48-days post-*c-MYC* induction were sacrificed for body and liver weight measurements. These livers were then snap-frozen for subsequent tissue analysis. Micro-MRI was performed in the Animal Imaging Core at Rangos Research Center using a Bruker BioSpec USR 7T/30 (Billerica, MA, USA). Cohorts of three female and three male mice of each genotype were imaged 17 and 36 days post-*c-MYC* induction to quantify hepatic neoplasia, measure tumor size, and monitor tumor burden.

### 4.3. Immunostaining

Freshly isolated liver and tumor tissues were fixed in 4% paraformaldehyde. The histology core at Rangos Research Center (Pittsburgh, PA, USA) prepared slides with anti-Ki67 and anti-F4/80. A biotin-conjugated secondary antibody was used with the Vectastain ABC Elite kit (Abcam, Cambridge, MA, USA) for signal detection, along with Shandon hematoxylin solution (Thermo Fisher Scientific, Waltham, MA, USA) for counterstaining. The controls were stained with secondary biotin or immunoglobulin (IgG). The indicators were quantified by counting positive cells in a gated section using five fields of vision, 56 randomly selected from each slide at 20× magnification [47]. Additionally, liver sections were stained with hematoxylin and eosin (H&E) for evaluation by Dr. Paul Monga of the Clinical Biospecimen Repository Processing Core (CBRPC) of the Pittsburgh Liver Research Center (PLRC; Pittsburgh, PA, USA), in order to determine tumor grade and other histopathological characteristics. Dr. Monga was blinded to the genotype and time point of the tissues. For further analysis, prepared H&E stains were also sent to the CBRPC for digital slide scanning using a VS200 scanner (Olympus, Shinjuku City, Tokyo, Japan).

### 4.4. Immunoblotting

Tissue lysates in buffer containing 0.05% NP-40, 50 mM NaCl, 0.5 mM EDTA, 50 mM Tris-HCl (pH 7.4), and 10 mM NAM with 1X EDTA-free protease inhibitors (Roche Life Sciences, Basel, Switzerland) were electrophoresed on Criterion SDS polyacrylamide gels (Bio-Rad, Hercules, CA, USA) and transferred to nitrocellulose membranes. The primary antibodies used were anti-*Sirt2* (ab67299; Abcam), anti-GAPDH (G8795; Sigma-Aldrich, St. Louis, MO, USA), anti-c-MYC (Cat # 5605; Cell Signaling, Danvers, MA, USA), anti-Alpha tubulin (Cat # 66031-1-Ig, Proteintech, Rosemont, IL, USA), and anti-Lamin A/C (Cat # 10298-1-AP; Proteintech). After incubation with 1:10,000 Goat anti-Rabbit-HRP IgG (STAR208P; Bio-Rad) or Goat anti-Mouse-HRP IgG secondary antibody (STAR207P; Bio-Rad), the blots were visualized with Clarity Max ECL (BioRad), scanned, and in some cases subjected to densitometric analysis using ImageJ software.

### 4.5. Preparation of Cytosolic and Nuclear Fractions

The nuclear and cytosolic fractions were prepared from snap frozen tissue with the Minute Cytosolic and Nuclear Extraction kit (Invent Biotechnologies, Plymouth, MN, USA). The fractions were prepared in buffer containing 0.05% NP-40, 50 mM NaCl, 0.5 mM EDTA, 50 mM Tris-HCl (pH 7.4), and 10 mM NAM with 1X EDTA-free protease inhibitors (Sigma-Aldrich). The nuclear marker Lamin A/C (Proteintech) and the cytoplasmic marker GAPDH (Sigma-Aldrich) were used to detect the purity of the fractions.

### 4.6. RNAseq

Liver pieces were collected and snap frozen in LN2. A mass of 10 mg of tissue was thawed in five volumes of RNAlater (Thermo Fisher Scientific), and the RNA was isolated using the RNeasy Mini Kit (Qiagen, Germantown, MD, USA). A cDNA library was prepared via RT-PCR using random hexamer primer mix (Thermo Fisher Scientific). The cDNA was sent to the Rangos Genomics Core for integrity and quality control. The samples were sent to NovoGene for Illumina sequencing (Sacramento, CA, USA). An adjusted *p*-value was generated for the RNA-seq study by multiplying standard *p*-values by the number of comparisons (defined as q-value ≤ 0.01 as the cutoff for significance), to control for false discoveries.

### 4.7. Statistical Analysis

All of the comparisons are represented on graphs with an unpaired Student’s *t* test (GraphPad Version 7);* *p*-value < 0.05, ** *p*-value < 0.01, and *** *p*-value < 0.001 were used as cutoffs for statistical significance. All graphs represent mean ± standard deviation (SD) or individual values with mean represented.

## 5. Conclusions

This study showed that *Sirt2* functions as a promotor of HCC in vivo, supporting previous studies performed in vitro. The major findings include overall restricted tumor growth using a novel transgenic approach and new evidence that c-MYC’s stability and localization to the nucleus are mediated by *Sirt2* deacetylation. Current knowledge regarding *Sirt2*’s apparent interaction with c-MYC is limited to studies that have been performed in vitro. Not only is it necessary to confirm this interaction in vivo, but further studies to establish whether this interaction is direct or indirect are necessary to better understand this mechanism. Additionally, *Sirt2*^−/−^ HCCs acquire fewer TAMs. In support of previous reports, this study confirmed significant upregulation of the major gluconeogenic regulator, *Pck1* in *Sirt2*^−/−^ HCC mice, shown in vivo for the first time. This implicates *Sirt2*’s involvement in HCC glucose metabolism, potentially shifting focus away from the promotion of MYC stability to the Warburg effect as the major mechanism to explain the tumor phenotype. This research was performed with the intent to evaluate the potential for *Sirt2* inhibition as a treatment option for HCC in vivo. This intention reflects broader goals within the biomedical space to develop therapeutics to help alleviate the individual and public health burdens carried by HCC. The research completed here adds to our current knowledge base regarding *Sirt2* biology and regulation of tumorigenesis, which is paramount for the development of new treatment options for those living with HCC.

## Data Availability

All relevant data are included in the manuscript or in the Supplemental Data files.

## Supplementary Materials

The following supporting information can be downloaded at: https://www.mdpi.com/article/10.3390/ijms241612618/s1.
